# Gaze tracking dataset for comparison of smooth and saccadic eye tracking

**DOI:** 10.1016/j.dib.2021.106730

**Published:** 2021-01-09

**Authors:** Adam Pantanowitz, Kimoon Kim, Chelsey Chewins, David M Rubin

**Affiliations:** School of Electrical & Information Engineering, University of the Witwatersrand, Johannesburg South Africa

**Keywords:** Eye movement, Saccadic eye tracking, Smooth eye tracking, Image processing, Pupil detection, Vestibulo-ocular reflex

## Abstract

Pupil tracking data are collected through the use of an infrared camera, and a head-mounted system [1]. The head-mounted system detects the relative pupil position and adjusts the mouse cursor position accordingly. The data are available for comparison of eye tracking with saccadic movements (with the head fixed in space) versus those from smooth movements (with the head moving in space). The analysis comprises two experiments for both types of eye tracking, which are performed with ten trials each for two participants. In the first experiment, the participant attempts to place the cursor into a target boundary of varying sizes. In the second experiment, the participant attempts to move the cursor to a target location within the shortest time.

## Specifications Table

**Table:** 

Subject	Human-Computer Interaction
Specific subject area	Eye tracking for controlling the position of a mouse cursor
Type of data	Table
How data were acquired	Data acquired from head-mounted camera (Sony Playstation-Eye camera, infrared light circuit, headgear) and computer software to detect pupil and control mouse
Data format	Raw Analysed Filtered
Parameters for data collection	Two of the authors tested the system by sitting upright on a chair reclined at a 100 ° angle. The chair was adjusted to ensure that both feet were flat on the ground. The computer monitor was positioned directly in front of them at eye level, at a distance of 50 cm. The user's head and eye position were kept constant at the beginning of each of the tests for both the smooth and the saccadic eye tracking experimentation.
Description of data collection	Data of the mouse cursor position for the various trials in each experiment were collected by storing the cursor position and the time of the trial.
Data source location	Institution: University of the Witwatersrand, Johannesburg City/Town/Region: Johannesburg Country: South Africa
Data accessibility	With the article
Related research article	Adam Pantanowitz, Kimoon Kim, Chelsey Chewins, Isabel N. K. Tollman, David M. Rubin, Addressing the eye-fixation problem in gaze tracking for human computer interface using the Vestibulo-ocular Reflex, Journal of Informatics in Medicine Unlocked. doi:10.1016/j.imu.2020.100488

## Value of the Data

• Data from measurements compare two types of eye tracking, *viz.* saccadic and smooth, for comparison in time and in converging on a given target area of various sizes.
• Data are useful for researchers comparing these two eye tracking techniques.
• Data may be reused for analysis of comparison of these types of eye tracking without building the system.

## 1 Data Description

### 1.1 Experiment 1

The objective of this experiment was to place the mouse cursor in a pixel area of a given box size.

The file, Experiment 1.xlsx, contains raw data with fields as per Table 1. Sheet “all” contains consolidated results for all the tests. Sheet “Summary” contains summarised results. Raw data are contained in remaining sheets, and are labelled according to the scheme: {participant} – {tracking type} – {target size}. “p1” and “p2” distinguish participants, “VOR” vs “saccadic” distinguish smooth vs saccadic tracking respectively, and “9”, “25”, “50”, “100”, distinguish the square length of the target box.

**Table 1:** Data description for Experiment 1 (*all sheet* of Experiment 1.xlsx; or Experiment 1.csv).

Field	Description
Tracking	Type of tracking, viz. saccadic (labelled “saccadic” and smooth (labelled “VOR”)
Trial	The experimental trial, for each experiment taken
Measurement	A sequential measurement identifier for the individual measurement
Target Size	The size (in pixels) of the target square into which the user attempted to place the mouse cursor
Time	The recorded time in milliseconds
x	The horizontal mouse position
y	The vertical mouse position
Participant	The participant recorded

Data are also included in csv (Experiment 1.csv).

### 1.2 Experiment 2

The objective of this experiment was to measure the time taken to move the cursor from one specified position to another objective point.

The file, Experiment 2.xlsx, contains raw data with fields as per Table 2. Sheet “all” contains consolidated results for all the tests. Sheet “Summary” contains summarised results. Raw data are contained in remaining sheets, and are labelled “p1” and “p2” to distinguish participants, and “VOR” vs “saccadic” to distinguish smooth vs saccadic tracking respectively.

**Table 2:** Data description for Experiment 2 (*all sheet* of Experiment 2.xlsx; or Experiment 2.csv).

Field	Description
Tracking	Type of tracking, viz. saccadic (labelled “saccadic” and smooth (labelled “VOR”)
Trial	The experimental trial, for each experiment taken
Measurement	A sequential measurement identifier for the individual measurement
Exact Time	The precise time at which the cursor is triggered to reach the destination position
Time	The recorded time in milliseconds
x	The horizontal mouse position
y	The vertical mouse position
Participant	The participant recorded (1 or 2)

Data are also included in csv (Experiment 2.csv).

The consolidated data are processed in python in analysis.py and plotting.py. Details of the experimental methods and post-processing are included in the subsequent section. Various experimental trial run pairs from Experiment 2 are plotted in the “runs” subfolder, producing a series of 42 figures. The figures are named as follows:

_#{saccadic tracking trial number}_#{smooth VOR tracking trial number}_#{experiment type}.png, where the experiment type is either “position-comparison” or “distance-tracking” for each trial.

## 2 Experimental Design, Materials and Methods

The system is divided into two subsystems: hardware and software.

### 2.1 Hardware subsystem

The hardware subsystem consists of the PlayStation-Eye camera, headgear, and infrared circuit. The system is based on a US Patent first proposed by Jahnke [2], is capable of tracking VOR-related smooth eye movements, and comprises a camera mounted on the user's head, allowing the user to remain focused on the cursor's position for tracking purposes.

With due consideration to safety, infrared provides a significant benefit to the user over visible light imaging, in that it does not distract the user, thus improving performance and reducing eye fatigue.

The headgear rig is a cricket (sport) helmet, modified to provide structural support for the PlayStation-Eye camera and the infrared circuit. A malleable copper wire is used to connect the PlayStation-Eye camera and infrared circuit to the headgear. This setup ensures that the camera and the IR LEDs are directed at the eye, as the head moves. This setup ensures that the imaging assembly comprising the camera and IR LEDs moves together with head movements in the case of smooth VOR-based tracking, while both the imaging assembly and the head remain fixed in the saccadic tracking system.

Pupil movement is acquired using a modified PlayStation-Eye camera attached to the headgear. The camera is modified to receive exclusively infrared light. The infrared circuit provides infrared light to the eyes. It consists of a micro-controller connected to a 3V source with two infrared LEDs connected to the micro-controller's pulse width modulation (PWM) pins. The 3V source is constructed by connecting two AA batteries, each of which has a nominal voltage of 1.5V, in series. The PWM duty cycle is set at 50% further to limit infrared exposure to the eyes for safety.

The PlayStation-Eye is connected to a computer via a Universal Serial Bus (USB). The Code Laboratories Eye (CL-Eye) platform driver, which is installed on the computer, provides a certified hardware driver so that third party software programs can access the PlayStation-Eye camera [3].

### 2.2 Software subsystem

The software subsystem is responsible for moving the mouse cursor once movement of the pupil has been detected. This is implemented using a C++ integrated development environment (IDE), such as Microsoft Visual Studio 2019, with OpenCV 4.0 for image processing (an open source C++ library). The mouse cursor was moved using the Windows.h library and the raw position data captured from the same library.

OpenCV 4.0 is downloaded from the website opencv.org, and the OpenCV library can be added to Visual Studio 2019 project using a guide by Park [4]. The video feed of the PlayStation-Eye camera is captured with OpenCV library using the VideoCapture function. This uses the default camera setting of the system from the installation of the Code Laboratories Eye (CL-Eye) platform driver.

In order to capture the mouse co-ordinates, the windows.h library is required which is inserted into the header of the code. Using this library, the current coordinates of the mouse cursor is captured using the GetCursorPos object. The GetCursorPos object is of class point which represents points in the cartesian co-ordinates. The GetCursorPos retrieves the current position of the cursor in an *x* and *y* pair of screen co-ordinates.

To move the mouse, the same windows.h library is utilised. There is, however, no built-in function to move the mouse cursor and therefore a custom function is created. The custom function called MouseMove is created, and it receives two parameters (the x and y co-ordinates) in order to move the mouse cursor. The MouseMove function gets the width of the screen using the GetSystemMetrics(SM_CXSCREEN) function, and height of the screen by using GetSystemMetrics(SM_CYSCREEN). It must be noted that Windows represent the cursor coordinates on the screen as a 16-bit value (2^16^–65535), therefore the destination co-ordinate to which the mouse is instructed to move must be normalised to these values. This is done by multiplying the x coordinates by 65535 and divided by the screen width (from the GetSystemMetrics(SM_CXSCREEN) function). The same is done for the screen height. These values are then can be used to move the mouse cursor using the SendInput function from the windows.h library.

When the system is initialised, the mouse cursor moves to the centre of the screen (960, 540). The user looks at the cursor so that the position of the pupil is captured for calibration purposes. The user presses the C key on the keyboard which captures the position of the pupil co-ordinates, and stores it in a separate CSV file using the ofstream object from the fstream library. This calibration is run each time before each experimental trial begins.

To record the cursor position, the GetCursorPos object is called at 334 ms intervals. The code opens the CSV file using the ofstream object from the fstream library and appends the time, x, and y coordinates in a CSV file for each time interval. There is no pre-processing or processing of data. Only the cursor position is captured. Once the data are captured, the data are saved, and the CSV file is then closed. When the next experimental trial takes place, the process is repeated, and the data are appended to the file. The experiment ends with the user pressing the escape key to stop the application. Before the application is stopped, the last measurements of time, x, and y co-ordinates are recorded by appending to the CSV, and the file is saved.

### 2.3 Methods

The authors tested the system by sitting upright on a chair reclined at a 100-degree angle. The chair was adjusted to ensure that both feet were flat on the ground. The computer monitor was positioned directly in front of the of them at eye level, at a distance of 50 cm.

The user's head and eye position were kept constant at the beginning of each of the tests for both the smooth and the saccadic eye tracking experimentation. This was done by asking the user to look at the centre of the computer monitor with their gaze normal to the screen before each test. This means that the position of the face and the eyes were assumed to be similar for both the smooth and the saccadic eye tracking experiments. Before conducting the tests, the custom-built calibration was used to calibrate the position of the pupil with the user's focus centre screen (indicated in the *Software* subsection by the use of the “C” keyboard button).

The users participated in two experiments. The first experiment was performed to measure accuracy and resolution by moving the cursor to a defined target pixel area of decreasing sizes. The repeatability of placing the cursor into one of these define boxes is a proxy for a measurement of precision of the overall system. The second experiment was performed to measure the time taken to move a mouse cursor from one starting position to a given destination.

For the first experiment, four squares were drawn with the following dimensions in pixels: 100 × 100; 50 × 50; 25 × 25; and 9 × 9. The aim was to move the cursor into the centre of each square. The squares were placed in the centre of the screen with coordinates (960,540). The user was required to move the cursor from the top-left corner, coordinates (0,0), to within the square. Both the smooth tracking and saccadic tracking systems were measured for comparison.

The second experiment measures the time taken for a user to move a mouse from one position to another. The cursor started at the position (100,500) and the users were required to move the cursor to a designated end point at x > 1700.

The analysis of the measurements are included in analysis.py and plotting.py, both of which contain *Python3* examples of how to analyse/process the data using *Pandas* [5] and present the data with *Seaborn*. In this *Python3* example code, trials are paired to create figures comparing saccadic tracking with the smooth VOR tracking system, producing various output images as described in the section Data Description.

The analysis in analysis.py aggregates the data with the intention of evaluating:

• From Experiment 1, the distance calculated from the final intended position, and the mean tracking time to reach the destination combined for both participants. The final distance is also corrected for by multiplying by 0.0276 cm/pixel, to determine the average final distance from the target in centimetres.
• From Experiment 2, the mean and standard deviation of the tracking time time across all trials for saccadic vs smooth VOR-based tracking for each participant and combined;

In the analysis, we compare the performance of each trial run of saccadic tracking vs smooth VOR-based tracking, with the objective of considering:

• The path from the start of the tracking to a given destination mouse cursor position.
• The time taken to track from the start to the end of the tracking of a given destination mouse cursor position.

## Ethics Statement

Informed consent was obtained (the users are authors and investigators in the study). Study performed with ethical clearance from the Human Research Ethics Committee (non-medical) at the University of the Witwatersrand, Johannesburg, clearance number: H19/09/42.

## CRediT Author Statement

**Adam Pantanowitz:** Conceptualisation, Methodology, Writing, Visualisation, Data Collation, Data Analysis, Supervision; **Kimoon Kim:** Metholodgy, Hardware, Software, Writing, Data Gathering & Analysis; **Chelsey Chewins:** Methodology, Hardware, Software, Writing, Data Gathering; **David M. Rubin:** Methodology, Data Analysis, Writing, Editing.

## Declaration of Competing Interest

The authors declare that they have no known competing financial interests or personal relationships which have or could be perceived to have influenced the work reported in this article.

## Appendix Supplementary materials

Image, application 1
