# Supplementary figures and images for: Gaze tracking dataset for comparison of smooth and saccadic eye tracking
Source: Data Brief. 2021 Jan 9;34:106730. doi: 10.1016/j.dib.2021.106730 (PMC7817423; doi:10.1016/j.dib.2021.106730)

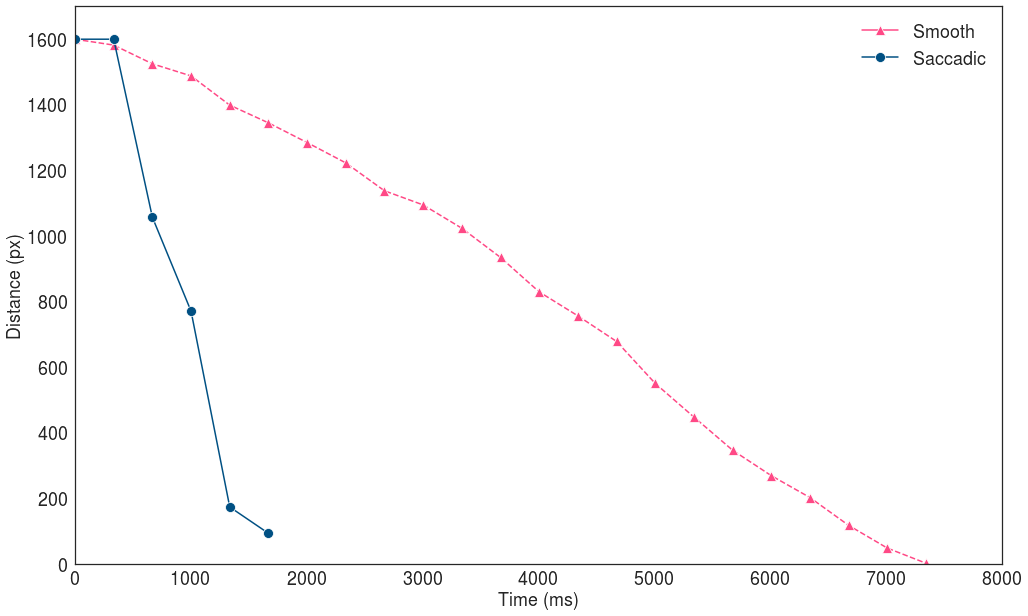

Supplement: Supplementary file 1 [file mmc1.zip › runs/_1_11_distance-tracking.png]

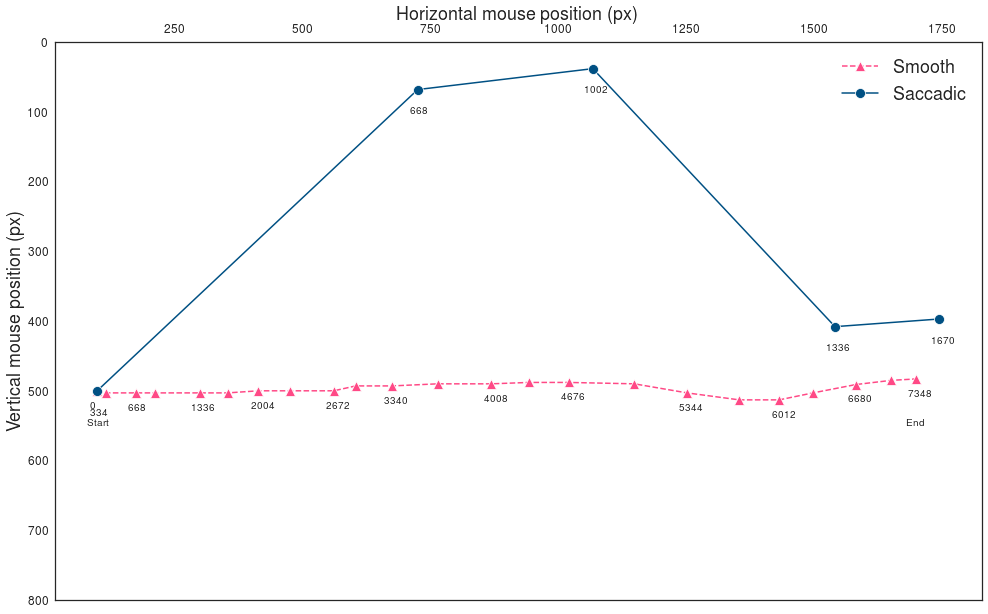

Supplement: Supplementary file 1 [file mmc1.zip › runs/_1_11_position-comparison.png]

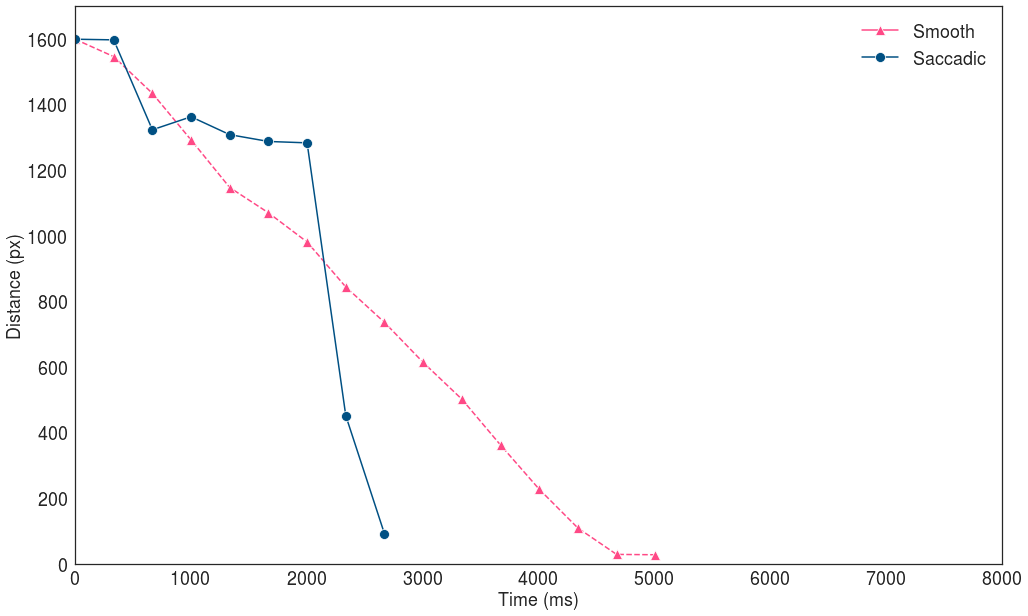

Supplement: Supplementary file 1 [file mmc1.zip › runs/_10_20_distance-tracking.png]

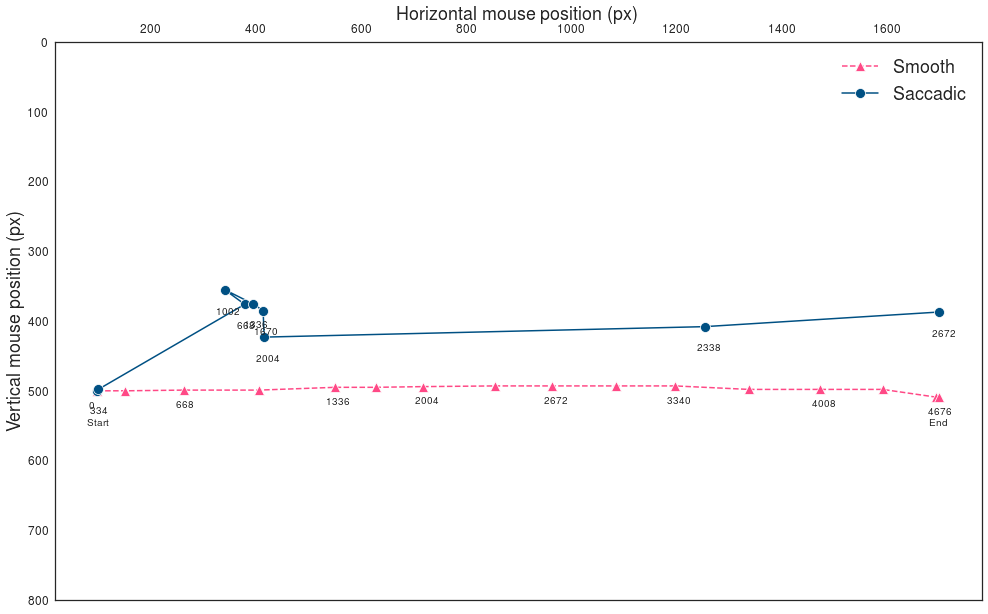

Supplement: Supplementary file 1 [file mmc1.zip › runs/_10_20_position-comparison.png]

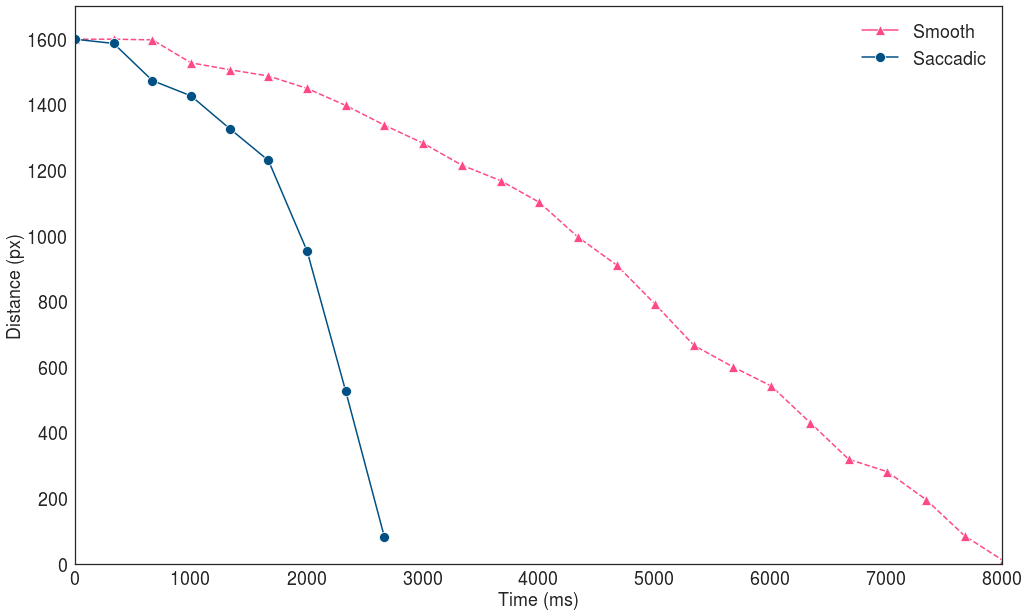

Supplement: Supplementary file 1 [file mmc1.zip › runs/_2_12_distance-tracking.png]

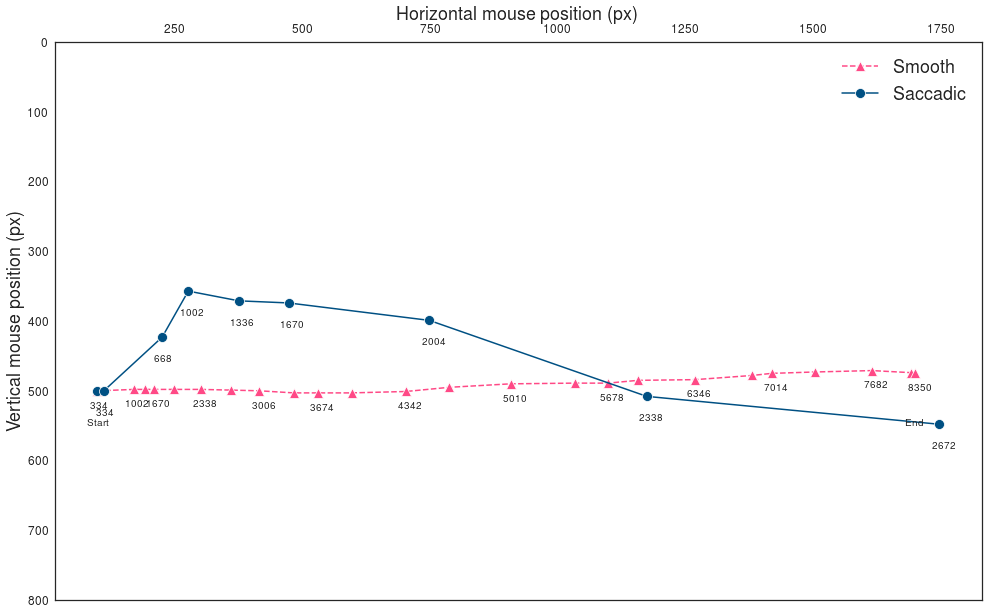

Supplement: Supplementary file 1 [file mmc1.zip › runs/_2_12_position-comparison.png]

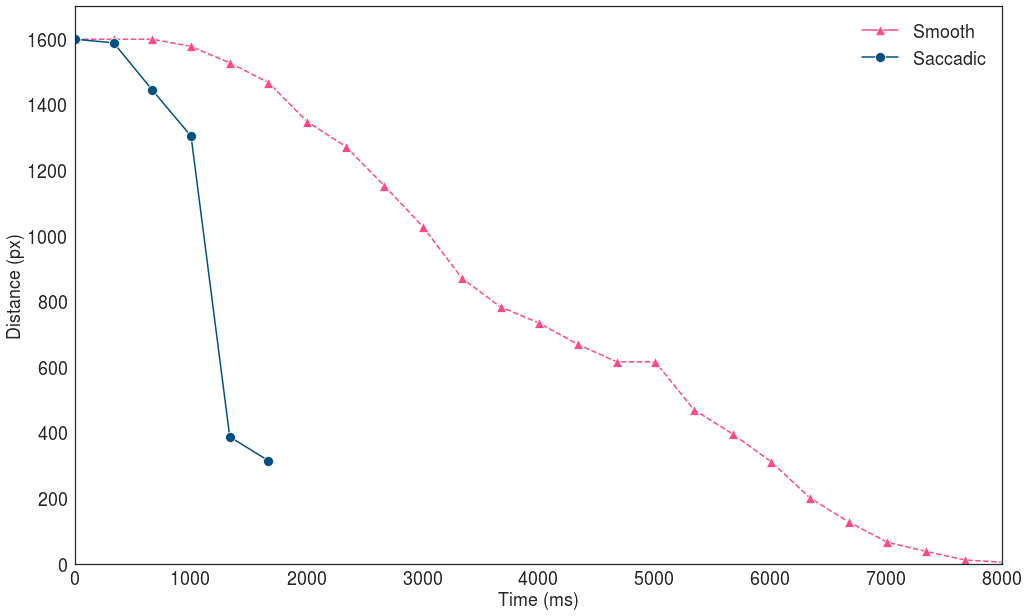

Supplement: Supplementary file 1 [file mmc1.zip › runs/_21_31_distance-tracking.png]

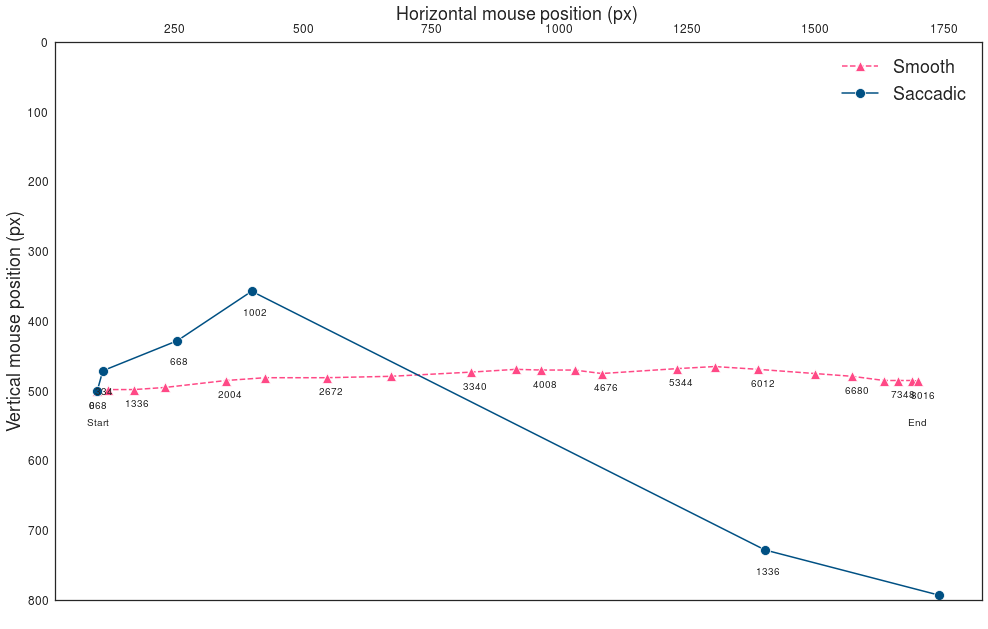

Supplement: Supplementary file 1 [file mmc1.zip › runs/_21_31_position-comparison.png]

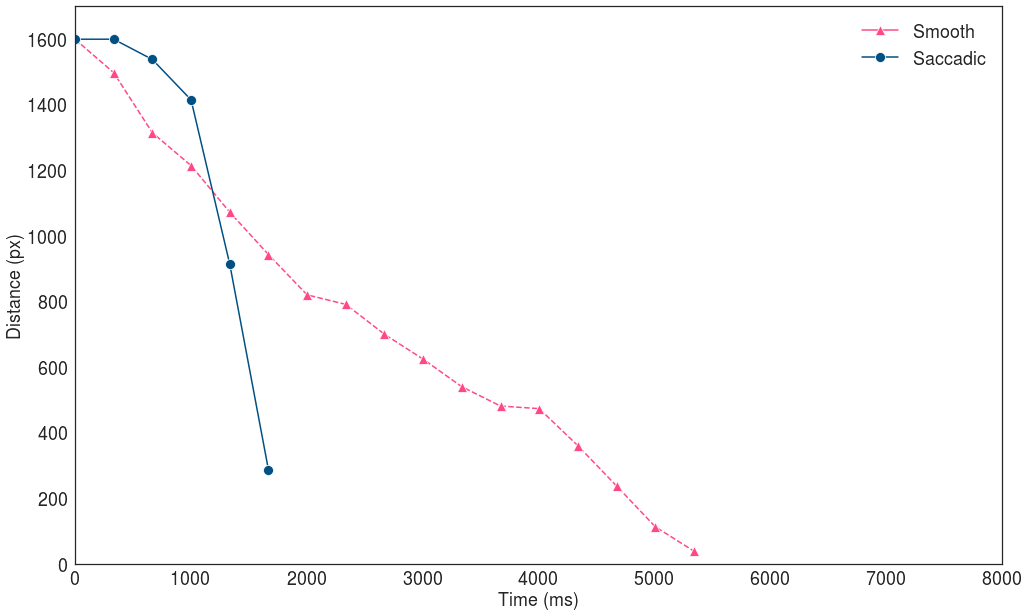

Supplement: Supplementary file 1 [file mmc1.zip › runs/_22_32_distance-tracking.png]

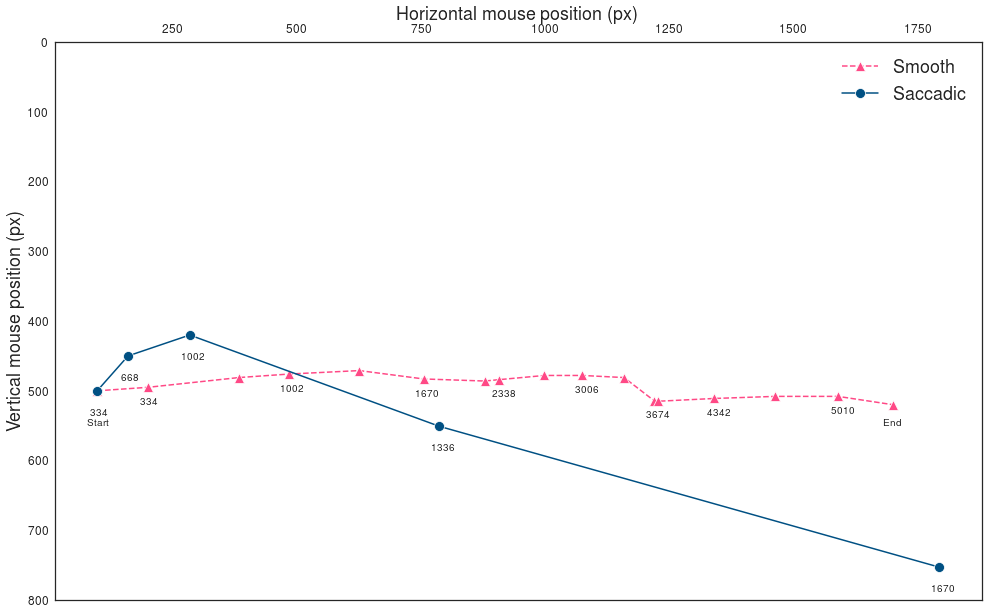

Supplement: Supplementary file 1 [file mmc1.zip › runs/_22_32_position-comparison.png]

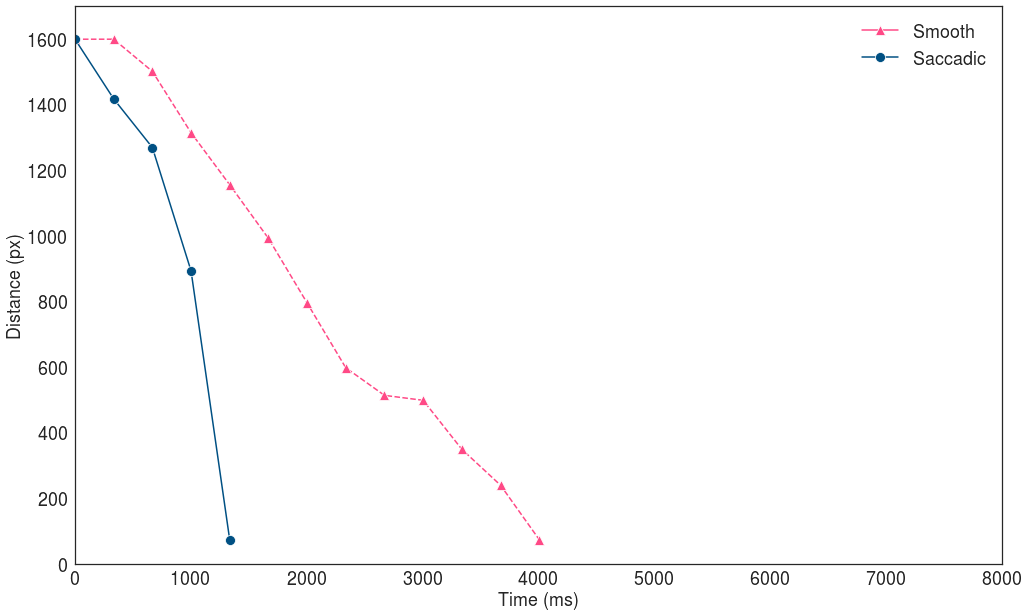

Supplement: Supplementary file 1 [file mmc1.zip › runs/_23_33_distance-tracking.png]

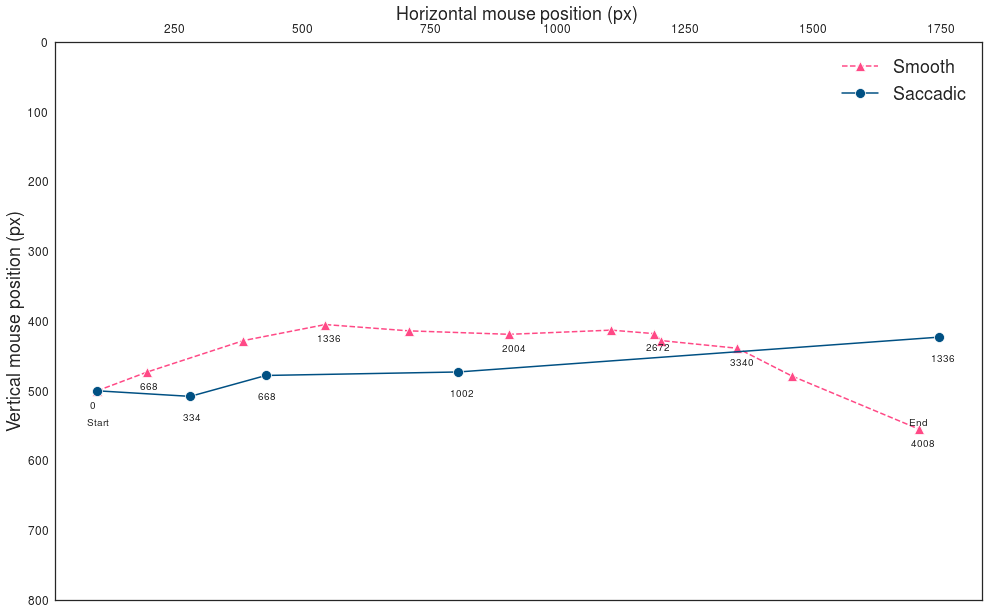

Supplement: Supplementary file 1 [file mmc1.zip › runs/_23_33_position-comparison.png]

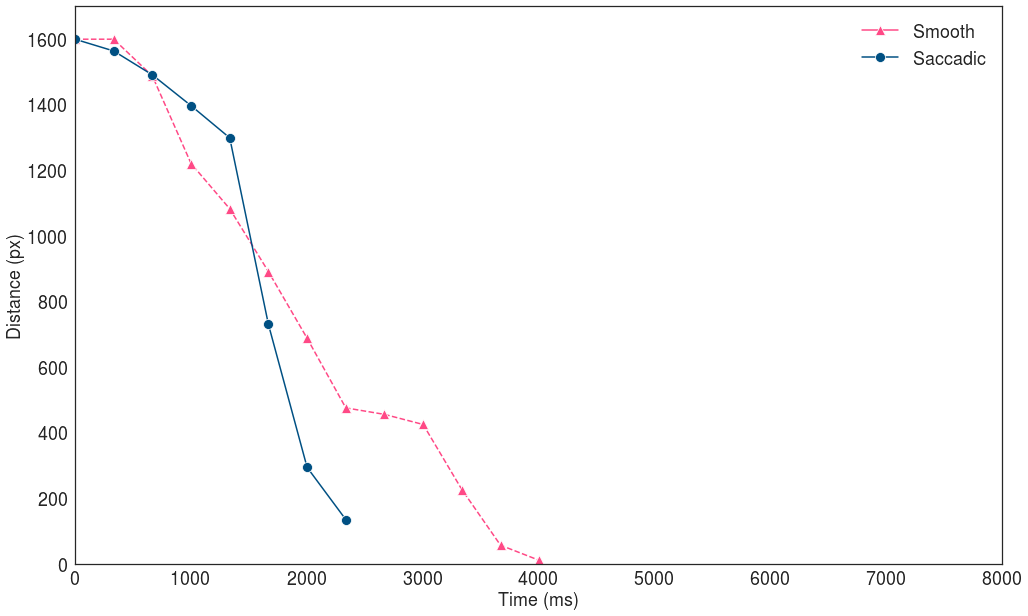

Supplement: Supplementary file 1 [file mmc1.zip › runs/_24_34_distance-tracking.png]

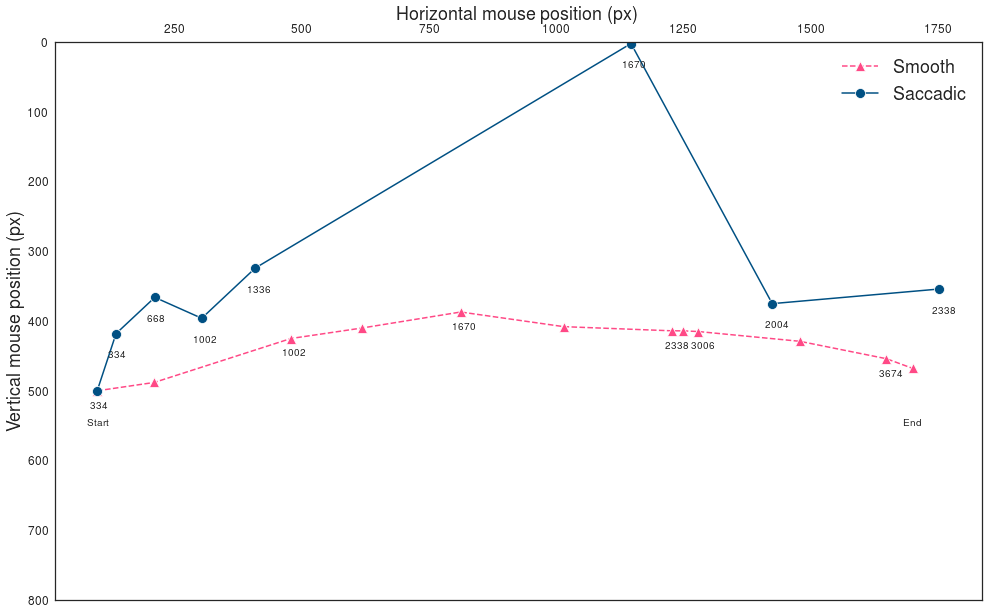

Supplement: Supplementary file 1 [file mmc1.zip › runs/_24_34_position-comparison.png]

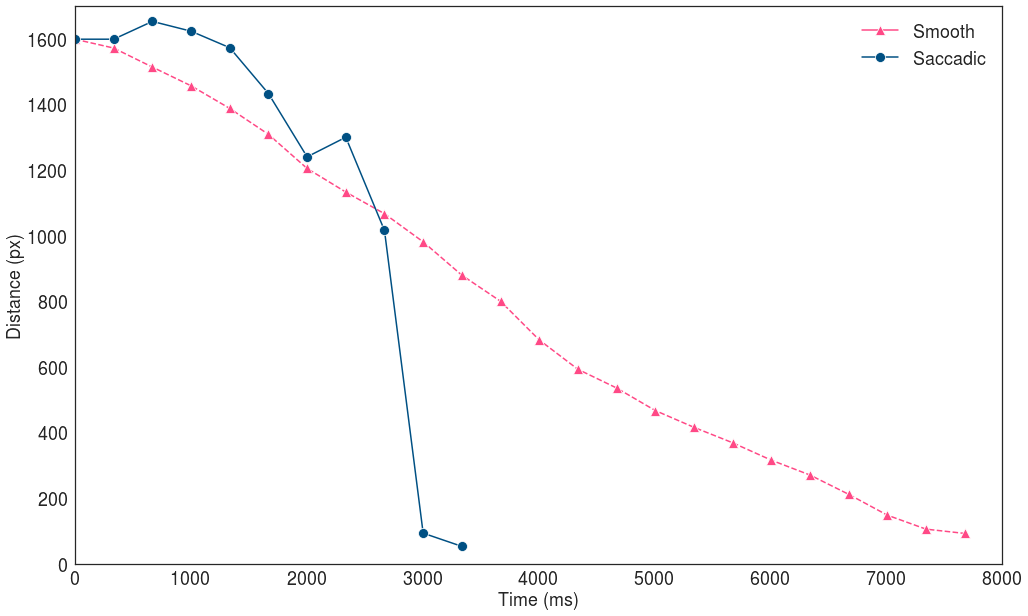

Supplement: Supplementary file 1 [file mmc1.zip › runs/_25_35_distance-tracking.png]

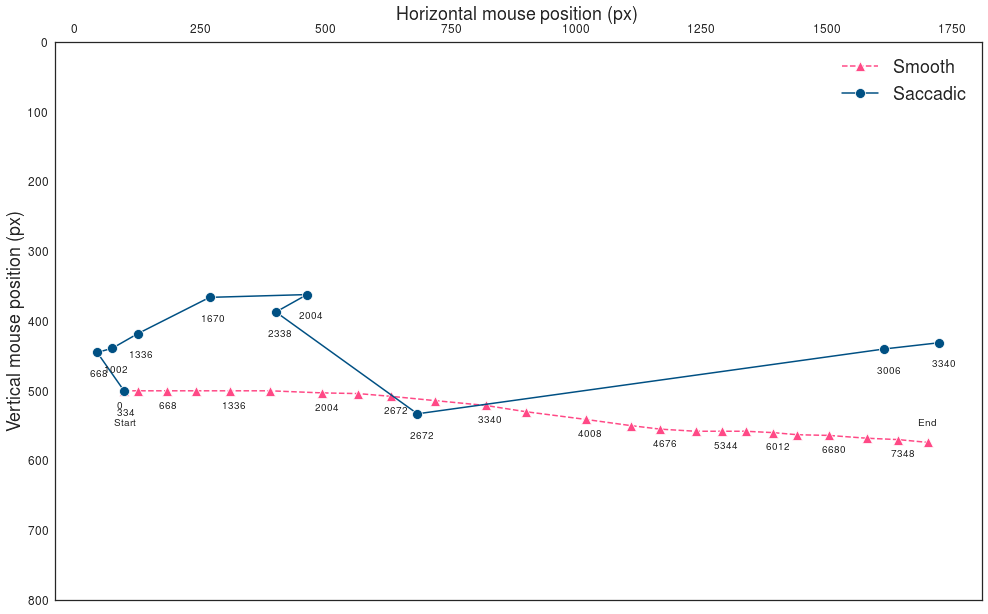

Supplement: Supplementary file 1 [file mmc1.zip › runs/_25_35_position-comparison.png]

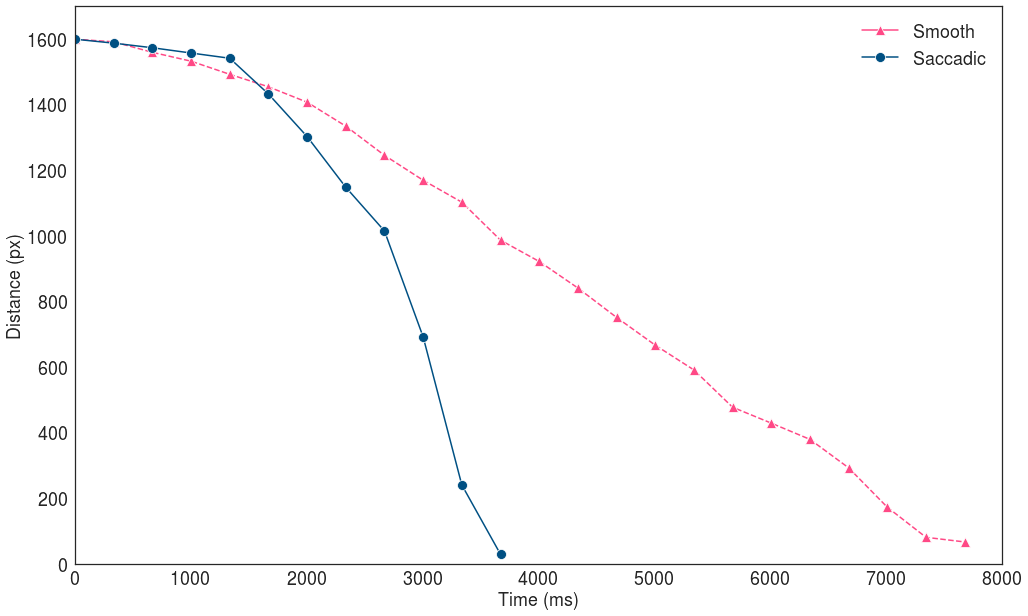

Supplement: Supplementary file 1 [file mmc1.zip › runs/_26_36_distance-tracking.png]

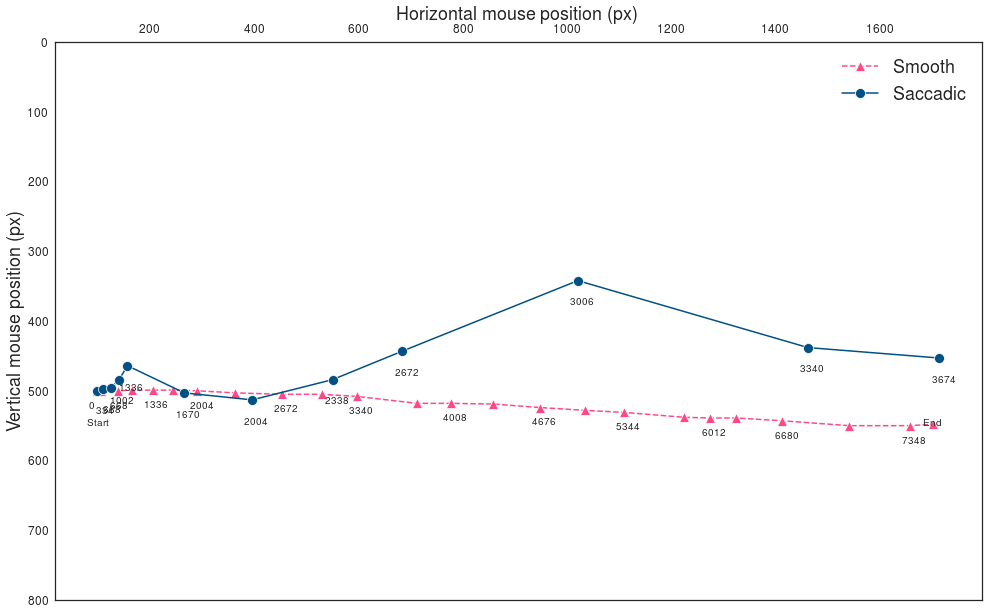

Supplement: Supplementary file 1 [file mmc1.zip › runs/_26_36_position-comparison.png]

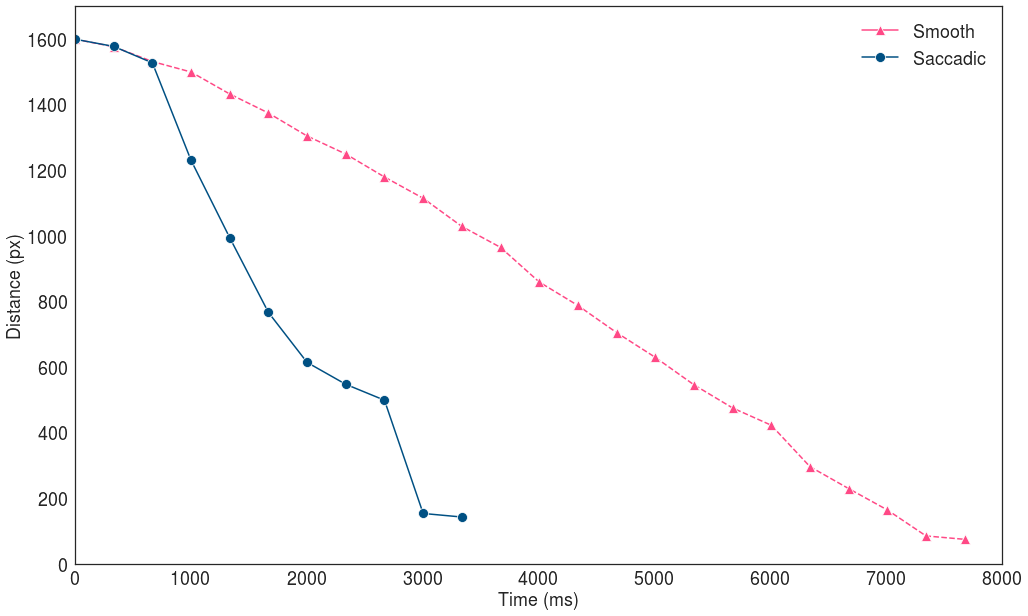

Supplement: Supplementary file 1 [file mmc1.zip › runs/_27_37_distance-tracking.png]

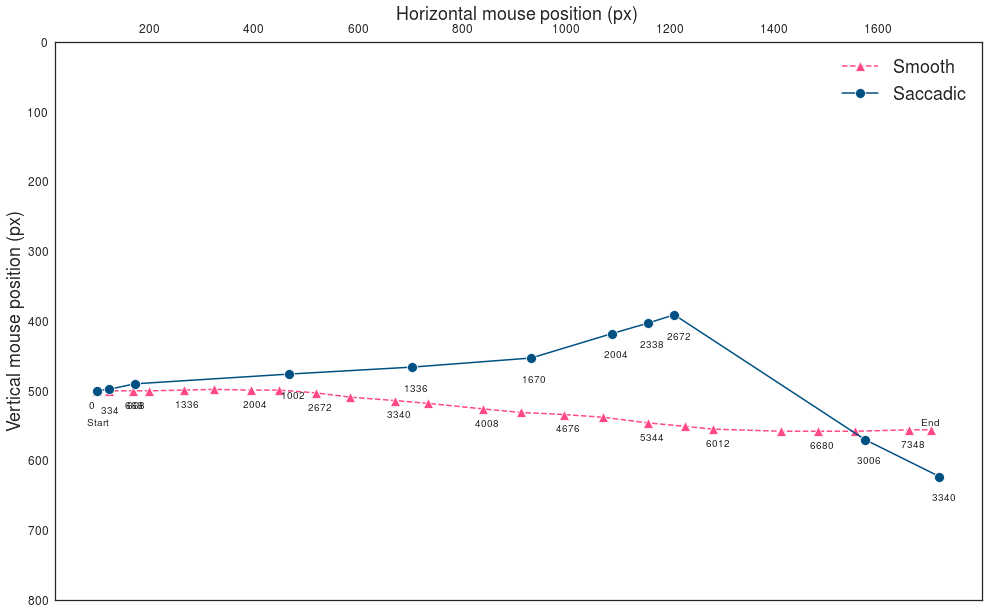

Supplement: Supplementary file 1 [file mmc1.zip › runs/_27_37_position-comparison.png]

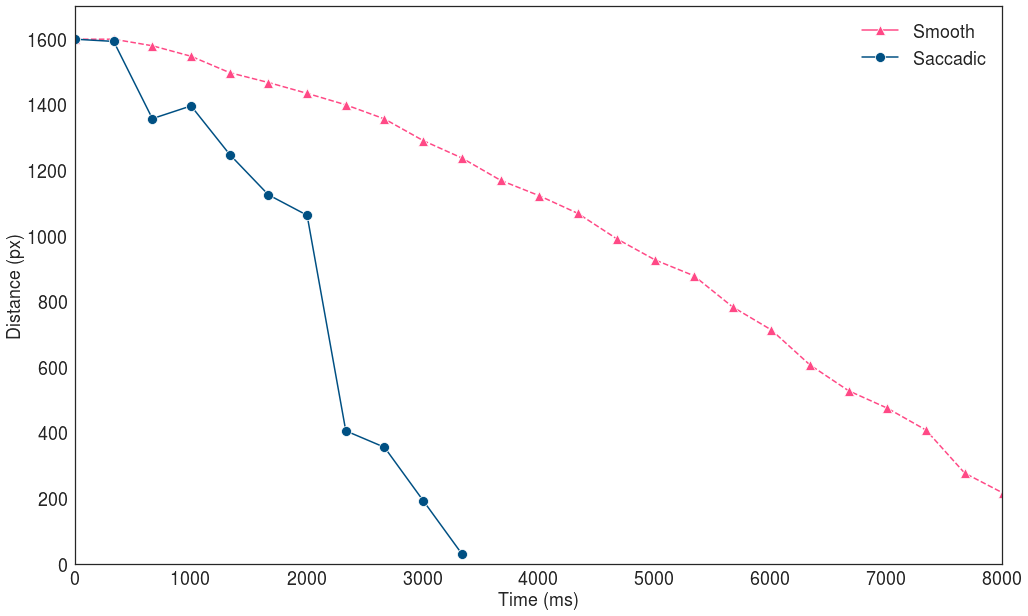

Supplement: Supplementary file 1 [file mmc1.zip › runs/_28_38_distance-tracking.png]

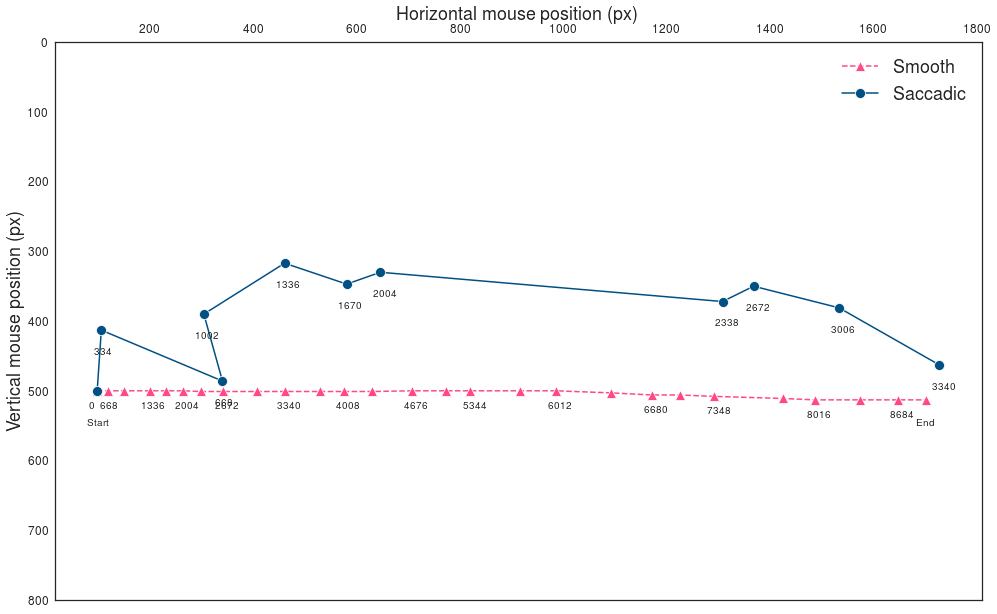

Supplement: Supplementary file 1 [file mmc1.zip › runs/_28_38_position-comparison.png]

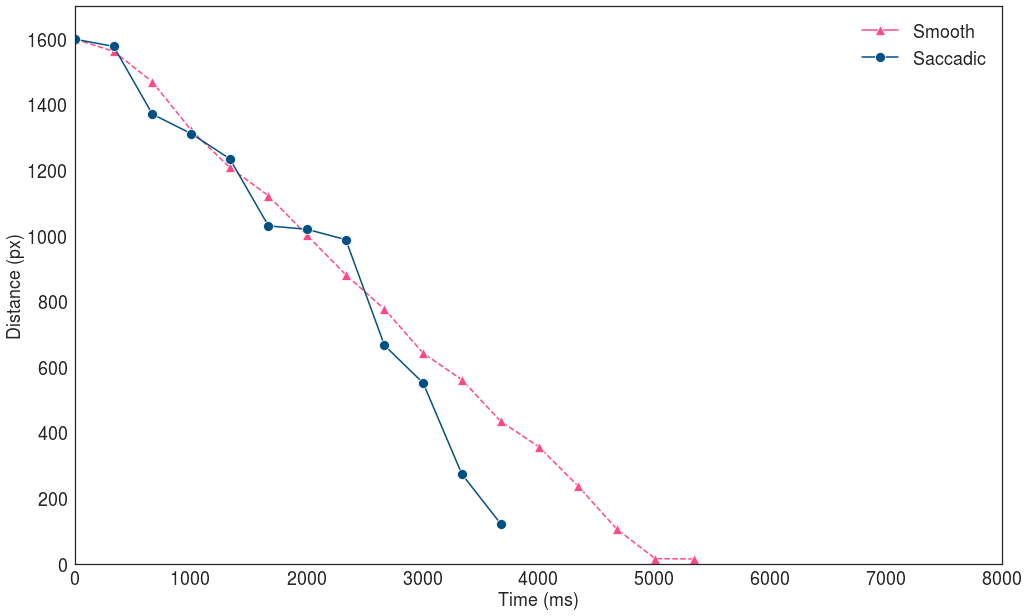

Supplement: Supplementary file 1 [file mmc1.zip › runs/_29_39_distance-tracking.png]

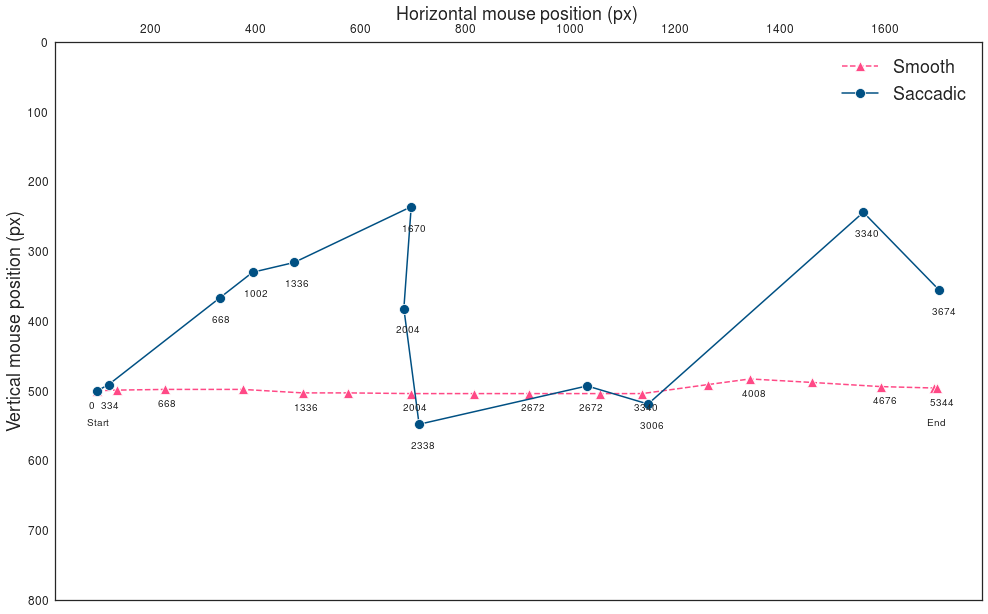

Supplement: Supplementary file 1 [file mmc1.zip › runs/_29_39_position-comparison.png]

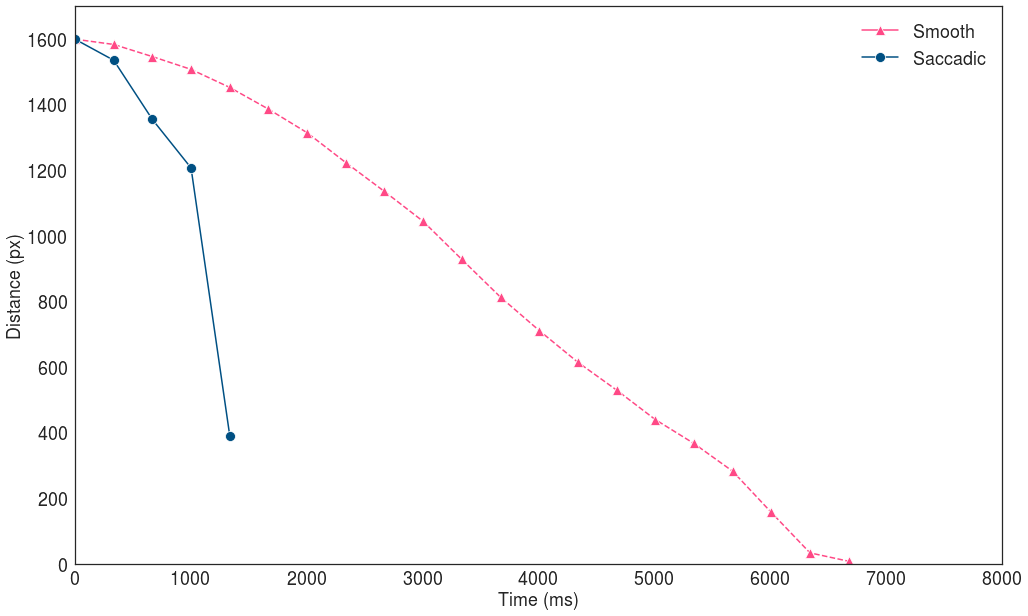

Supplement: Supplementary file 1 [file mmc1.zip › runs/_3_13_distance-tracking.png]

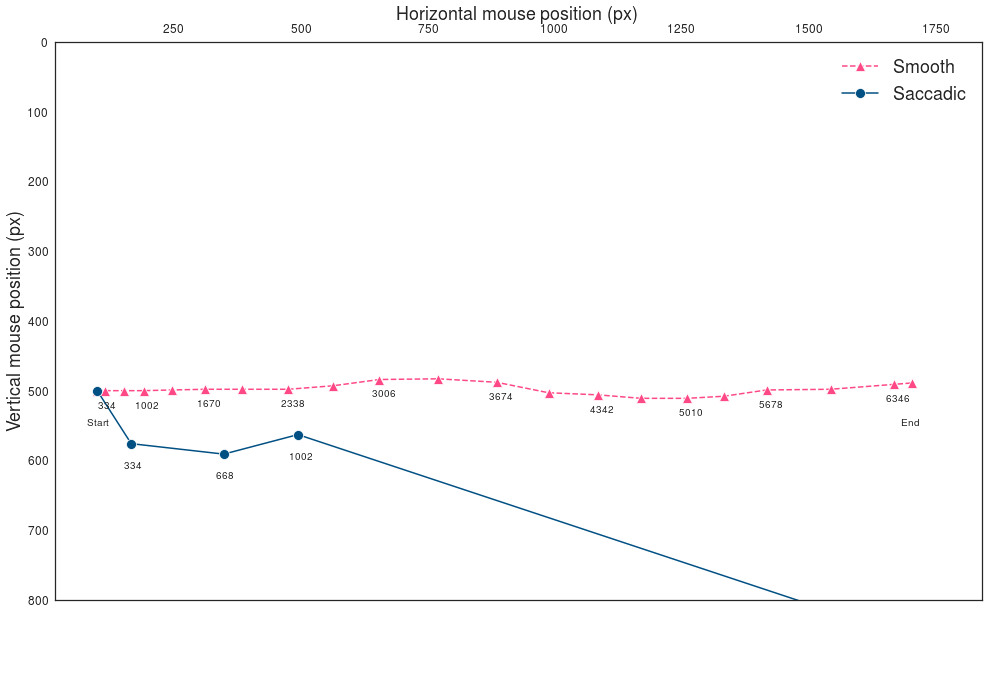

Supplement: Supplementary file 1 [file mmc1.zip › runs/_3_13_position-comparison.png]

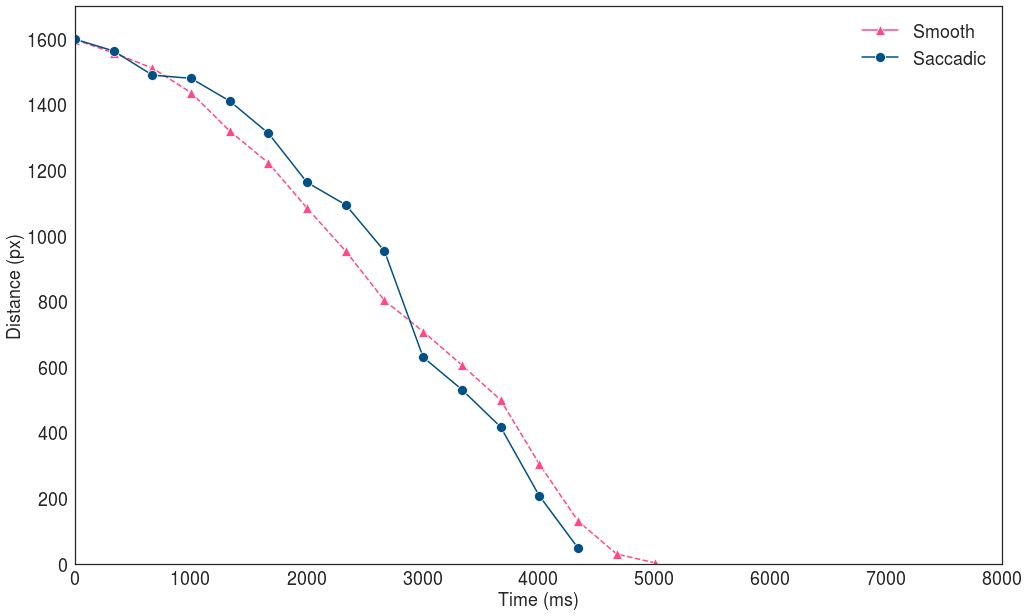

Supplement: Supplementary file 1 [file mmc1.zip › runs/_30_40_distance-tracking.png]

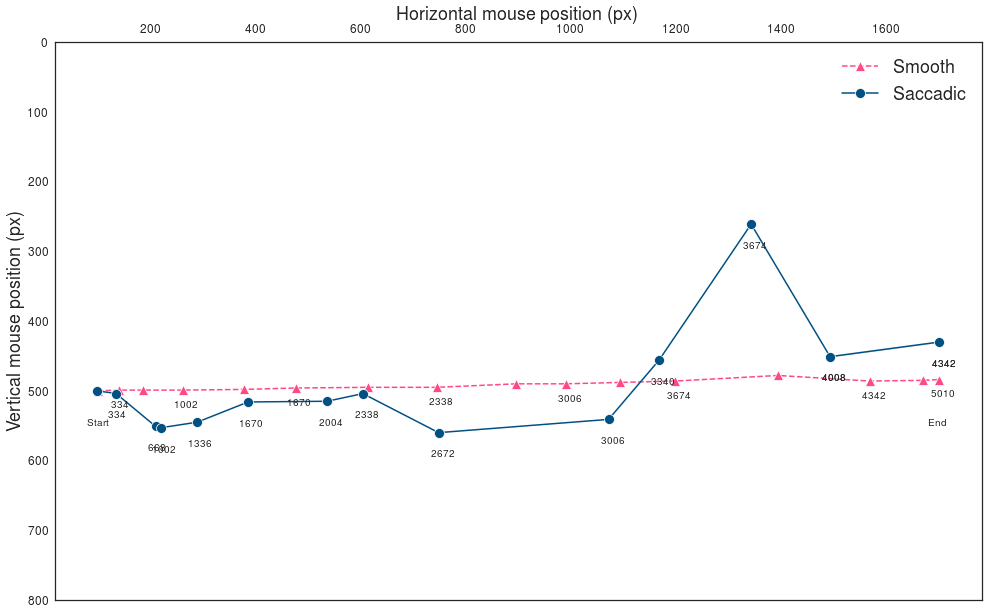

Supplement: Supplementary file 1 [file mmc1.zip › runs/_30_40_position-comparison.png]

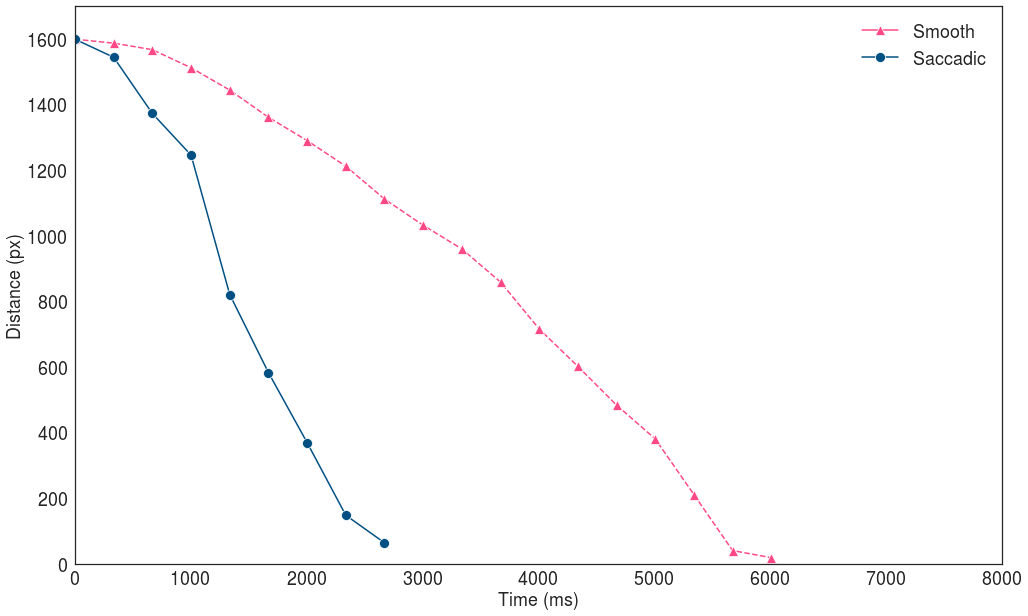

Supplement: Supplementary file 1 [file mmc1.zip › runs/_4_14_distance-tracking.png]

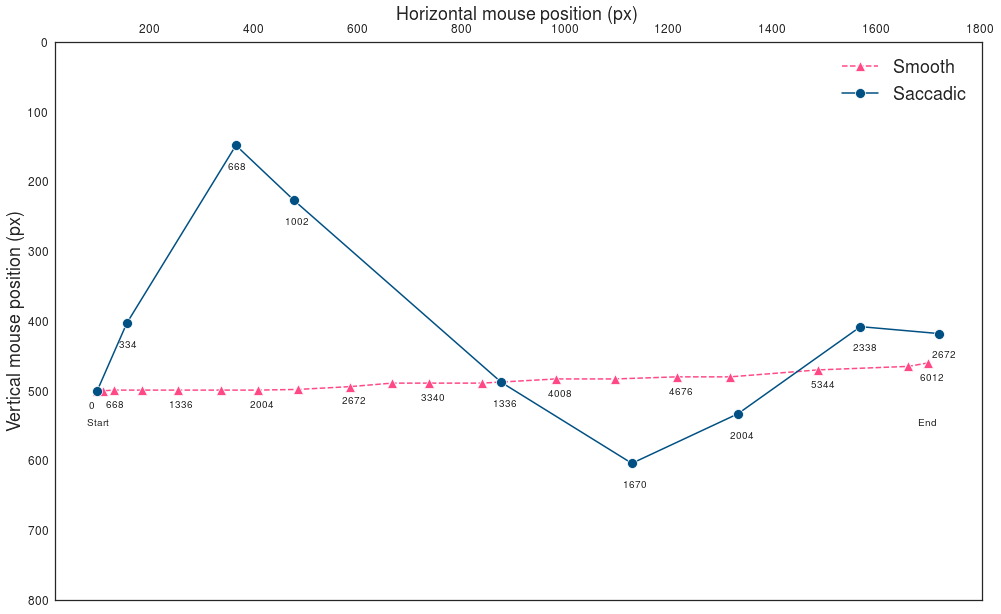

Supplement: Supplementary file 1 [file mmc1.zip › runs/_4_14_position-comparison.png]

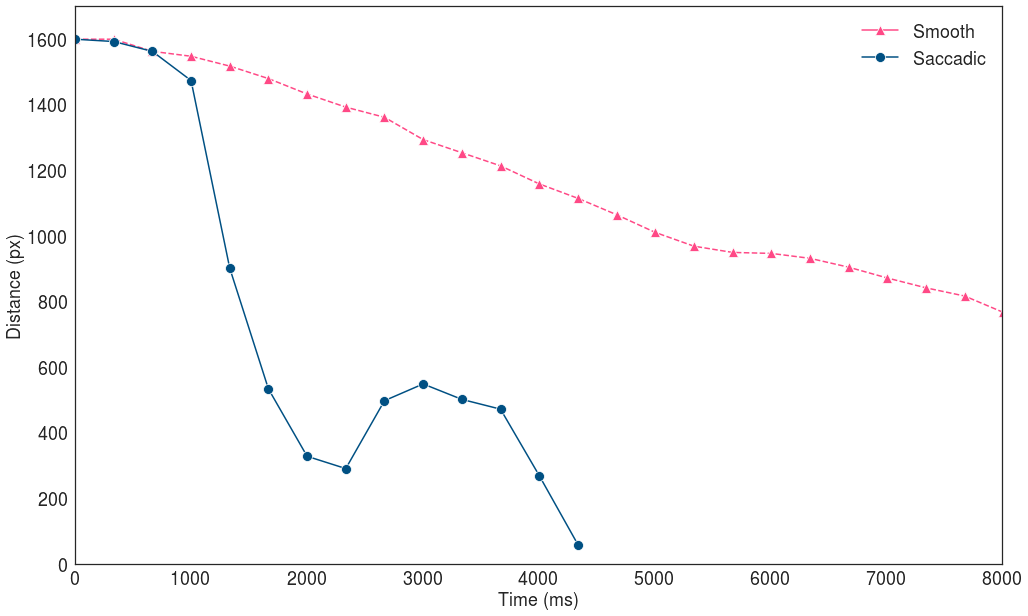

Supplement: Supplementary file 1 [file mmc1.zip › runs/_5_15_distance-tracking.png]

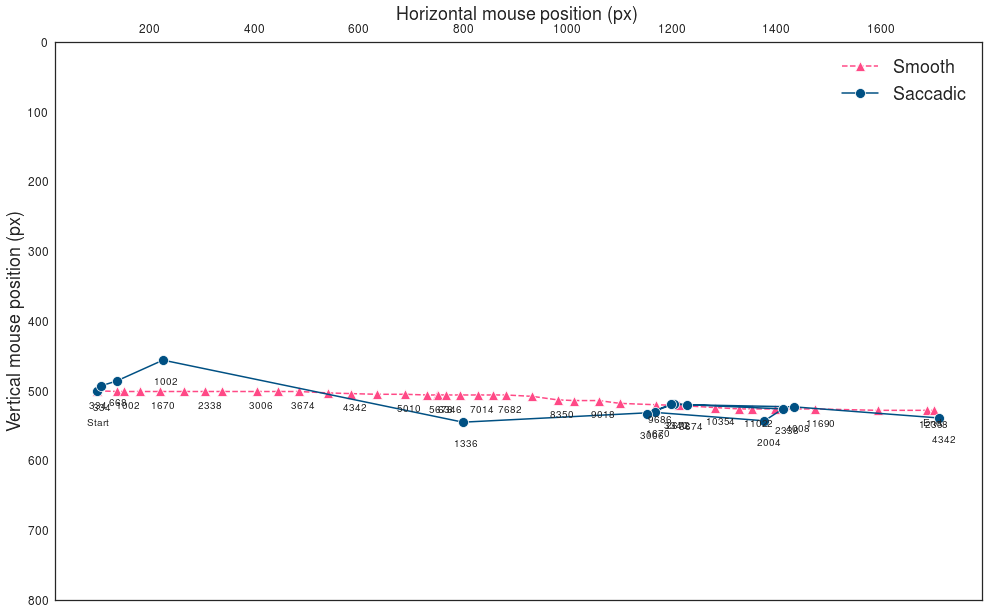

Supplement: Supplementary file 1 [file mmc1.zip › runs/_5_15_position-comparison.png]

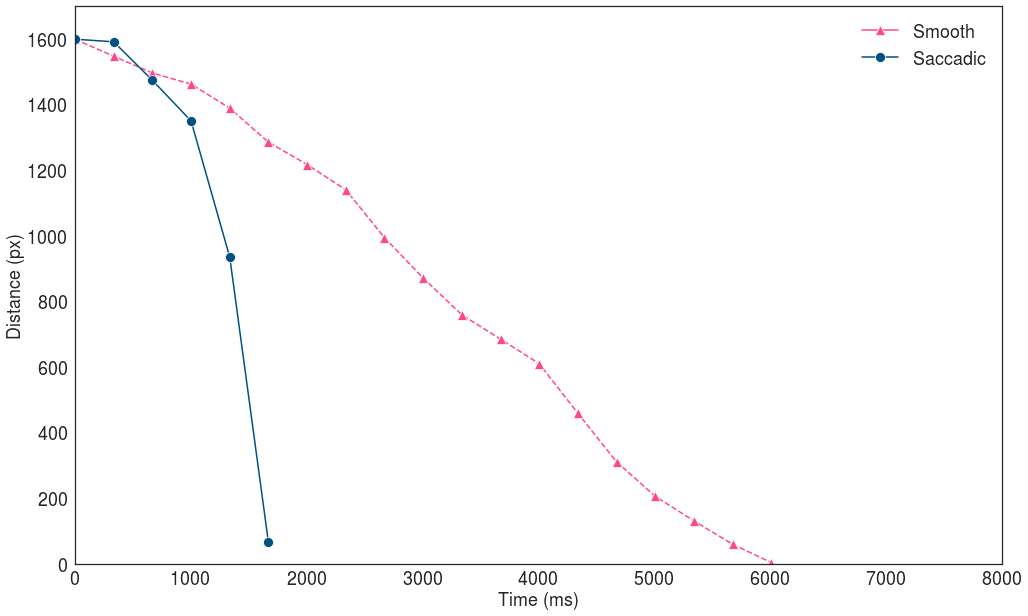

Supplement: Supplementary file 1 [file mmc1.zip › runs/_6_16_distance-tracking.png]

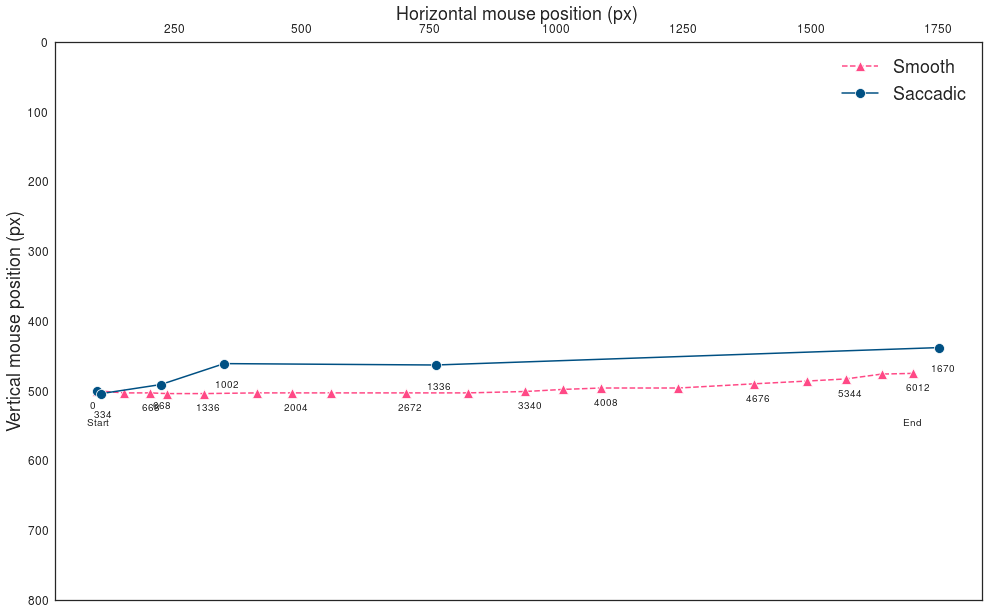

Supplement: Supplementary file 1 [file mmc1.zip › runs/_6_16_position-comparison.png]

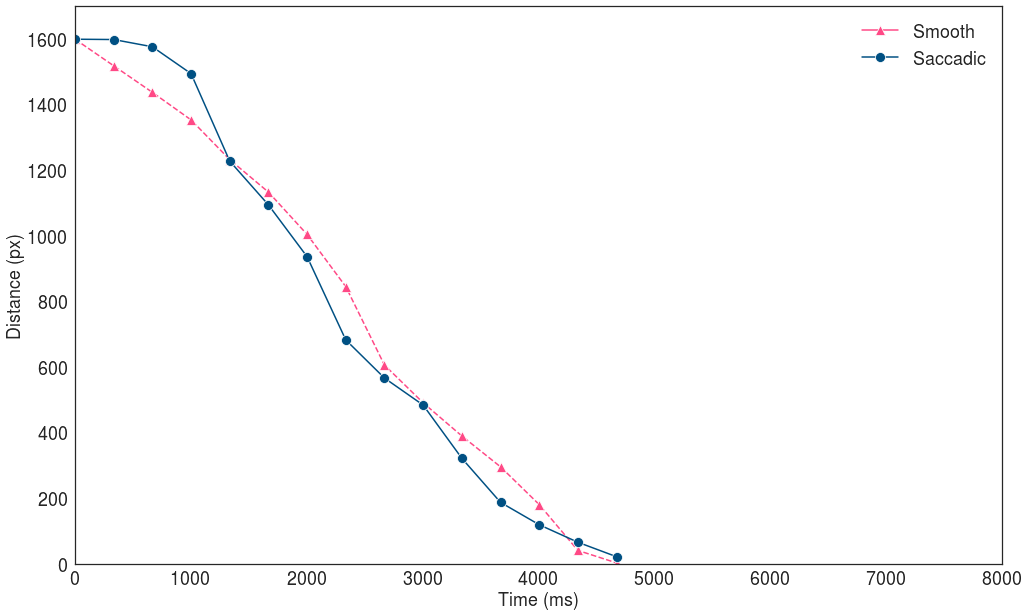

Supplement: Supplementary file 1 [file mmc1.zip › runs/_7_17_distance-tracking.png]

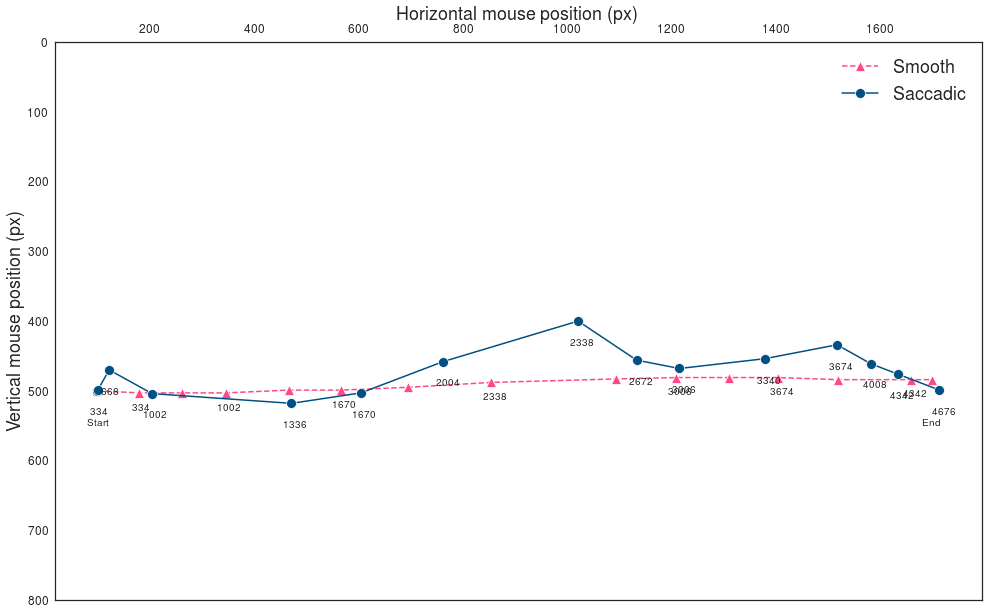

Supplement: Supplementary file 1 [file mmc1.zip › runs/_7_17_position-comparison.png]

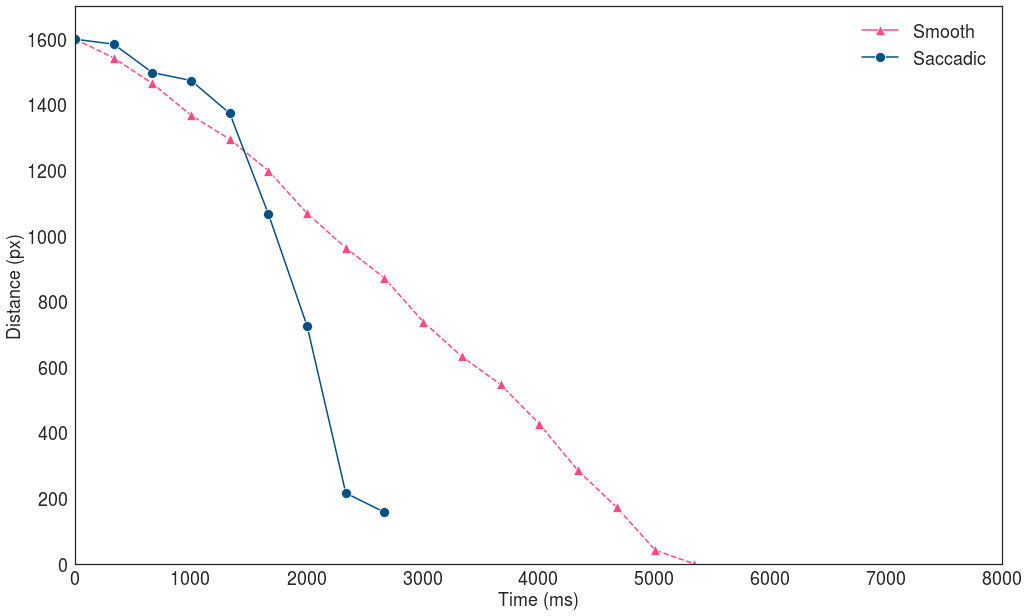

Supplement: Supplementary file 1 [file mmc1.zip › runs/_8_18_distance-tracking.png]

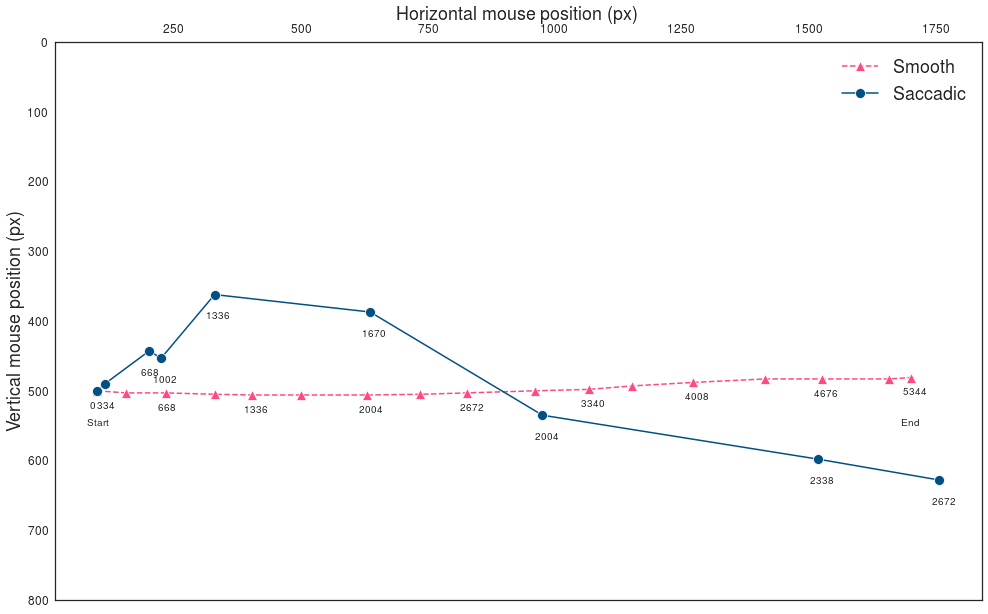

Supplement: Supplementary file 1 [file mmc1.zip › runs/_8_18_position-comparison.png]

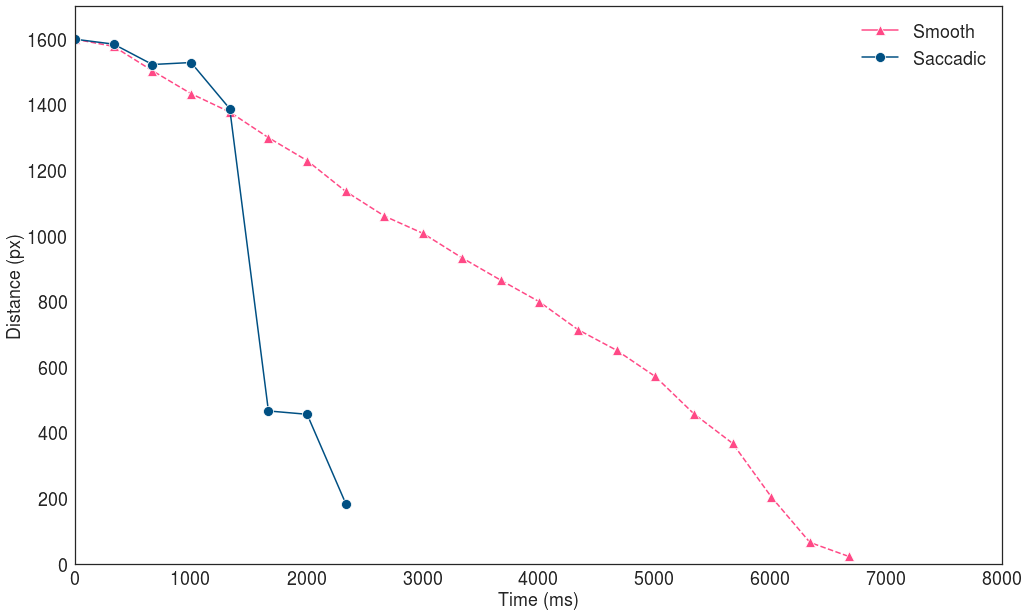

Supplement: Supplementary file 1 [file mmc1.zip › runs/_9_19_distance-tracking.png]

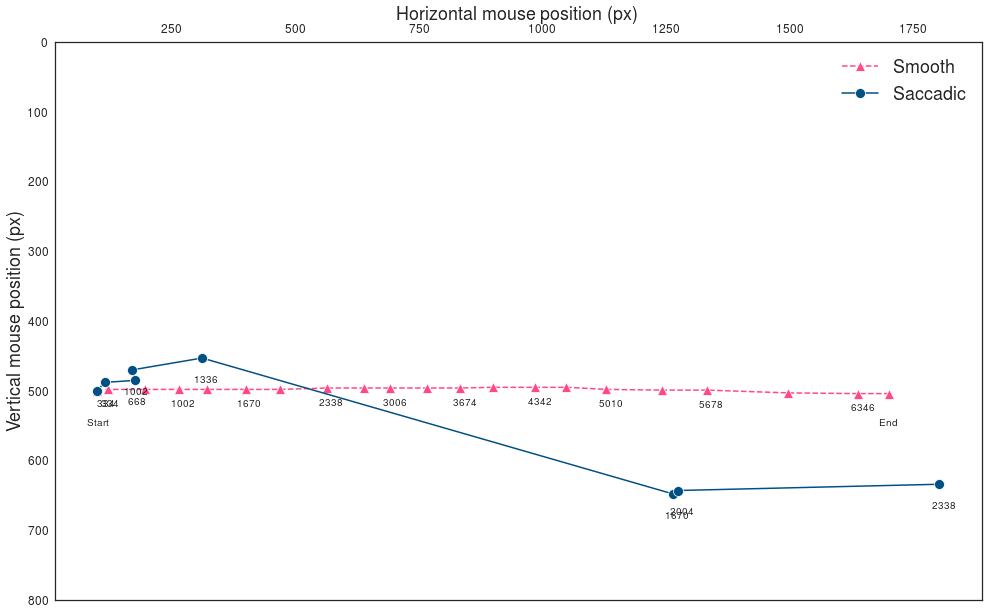

Supplement: Supplementary file 1 [file mmc1.zip › runs/_9_19_position-comparison.png]
